# Diffusion Tensor Imaging Analysis of Fine Motor Dysfunction and Recovery Following Cranioplasty

**DOI:** 10.7759/cureus.78510

**Published:** 2025-02-04

**Authors:** Yasuhiro Imada, Takashi Ueguchi, Kazuya Nakashima, Masato Hayashi, Yasushi Miura

**Affiliations:** 1 Department of Rehabilitation, Kobe Ekisaikai Hospital, Hyogo, JPN; 2 Department of Rehabilitation Science, Kobe University Graduate School of Health Sciences, Hyogo, JPN; 3 Center for Information and Neural Networks, Advanced ICT Research Institute, National Institute of Information and Communications Technology, Osaka, JPN; 4 Department of Radiation Oncology, Osaka University Graduate School of Medicine, Osaka, JPN; 5 Department of Medical Imaging Science, Osaka University Graduate School of Medicine, Osaka, JPN; 6 Department of Neurosurgery, Kobe Ekisaikai Hospital, Hyogo, JPN

**Keywords:** cranioplasty, diffusion tensor imaging (dti), fine motor function, magnetic resonance imaging (mri), traumatic brain injury (tbi)

## Abstract

We report a case of a 42-year-old woman who developed fine motor dysfunction after decompressive craniectomy for traumatic brain injury (TBI), despite the absence of obvious lesions on conventional magnetic resonance imaging (MRI) to explain the motor deficits. Following cranioplasty, diffusion tensor imaging (DTI) revealed an increase in fractional anisotropy (FA) in the primary motor cortex, which correlated with significant improvement in motor function. These findings highlight DTI's potential as a valuable tool for capturing subtle brain changes not apparent on conventional imaging techniques.

## Introduction

Diffusion tensor imaging (DTI) is an advanced modality of magnetic resonance imaging (MRI) that extends the capabilities of diffusion-weighted imaging (DWI). DWI measures water diffusion signals, and DTI utilizes data from multiple diffusion directions to map the three-dimensional diffusion of water molecules in the brain, enabling the evaluation of its microstructural organization. Key metrics derived from DTI include fractional anisotropy (FA), which reflects the integrity of white matter microstructure; mean diffusivity (MD), which indicates the magnitude of overall water diffusion and is associated with cellular density and extracellular space; and radial diffusivity (RD), which represents diffusion perpendicular to axonal fibers and is linked to myelin condition [1]. DTI has been applied in the field of neurorehabilitation, with studies reporting its utility in predicting motor and functional recovery after stroke and traumatic brain injury (TBI) based on white matter analysis [2-4]. Additionally, DTI has been used to investigate white matter changes in neurodegenerative diseases [5-7] and offers a quantitative approach to evaluate subtle microstructural changes that are difficult to detect with conventional MRI [8,9].

In this case report, we detail a patient with TBI complicated by brain herniation, where conventional imaging did not reveal any lesions associated with motor or sensory dysfunctions. However, the patient exhibited upper limb motor deficits, which improved following cranioplasty. To investigate the mechanisms underlying these motor impairments, we analyzed pre- and post-cranioplasty DTI data. Written informed consent for participation in this study was obtained from the patient.

## Case presentation

**Figure 1:**
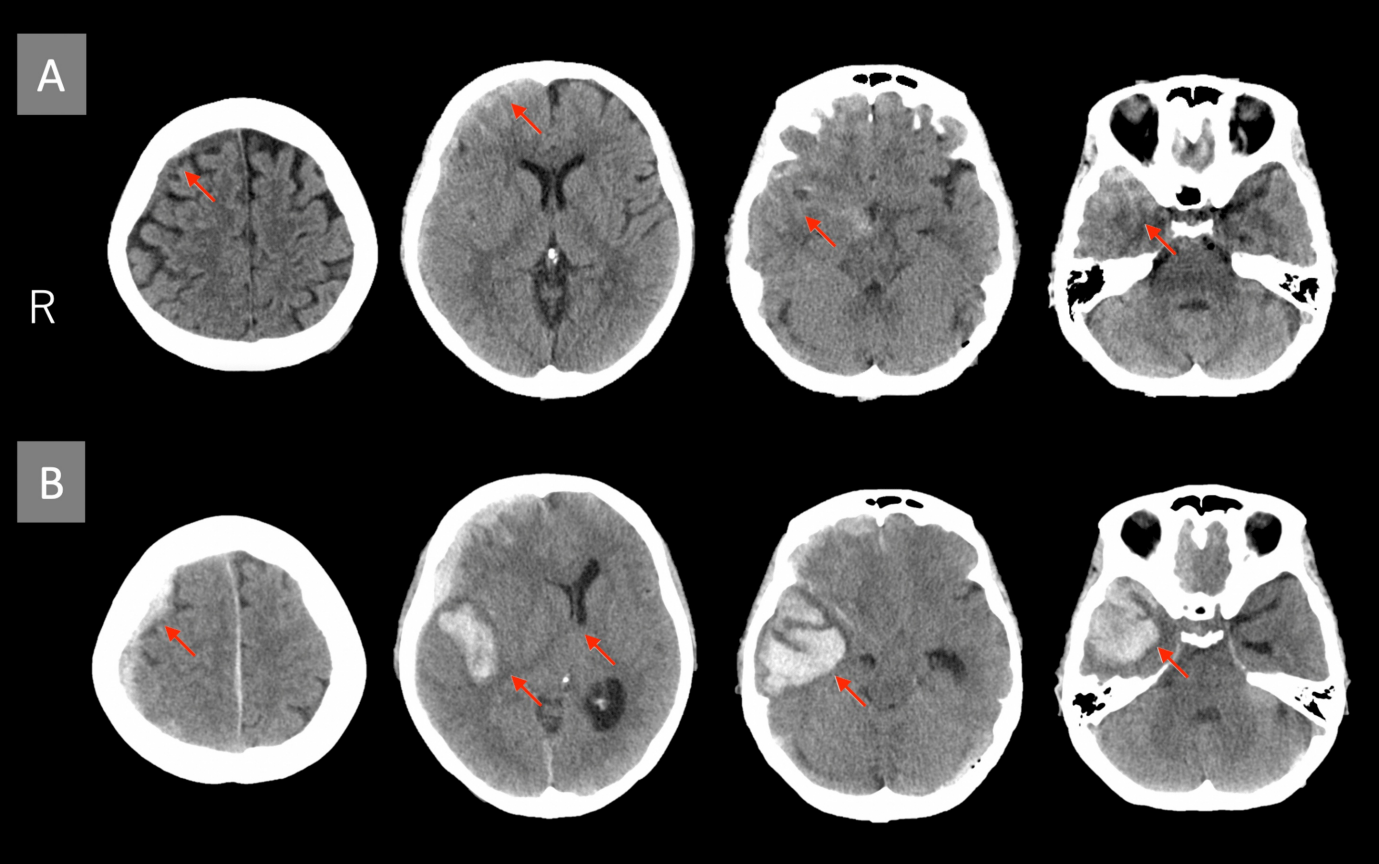
Initial and follow-up head CT scans (A) Initial head CT showing traumatic subarachnoid hemorrhage and acute subdural hematoma (arrows). (B) Follow-up head CT showing a hematoma in the right temporal lobe with midline shift (arrows). CT: computed tomography

A 42-year-old, right-handed woman sustained a head injury after falling down the stairs following alcohol consumption. She had no history of neurological or musculoskeletal conditions that could affect motor function. Initially, she presented neither an altered level of consciousness nor nausea. After she was transported to the hospital by emergency services, a head computed tomography (CT) scan revealed traumatic subarachnoid hemorrhage and acute subdural hematoma, leading to her hospitalization for conservative management (Figure 1A). Approximately eight hours later, the patient developed altered consciousness. A subsequent head CT scan revealed a hematoma in the right temporal lobe with a midline shift (Figure 1B). Consequently, decompressive craniectomy (internal and external decompression) was performed on the second day of hospitalization, involving partial resection of the right temporal lobe and removal of the temporal bone.

**Figure 2:**
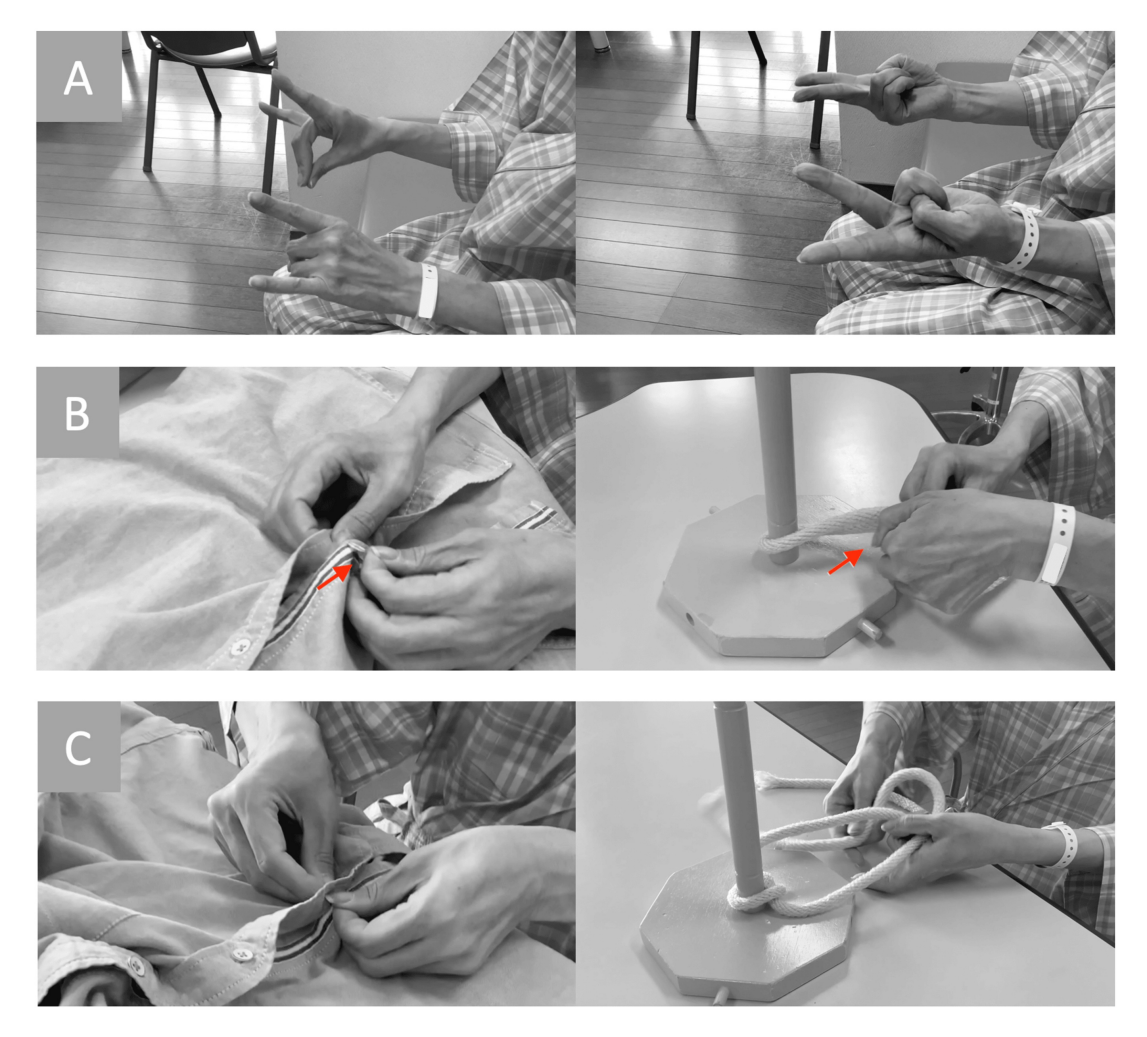
Motor function of the patient's fingers (A) Pre-cranioplasty imitation tasks: no evidence of fine motor dysfunction. (B) Pre-cranioplasty fine motor tasks: time-consuming performance, with difficulty grasping rope or buttons (arrows). (C) Post-cranioplasty fine motor tasks: no evidence of fine motor dysfunction.

**Figure 3:**
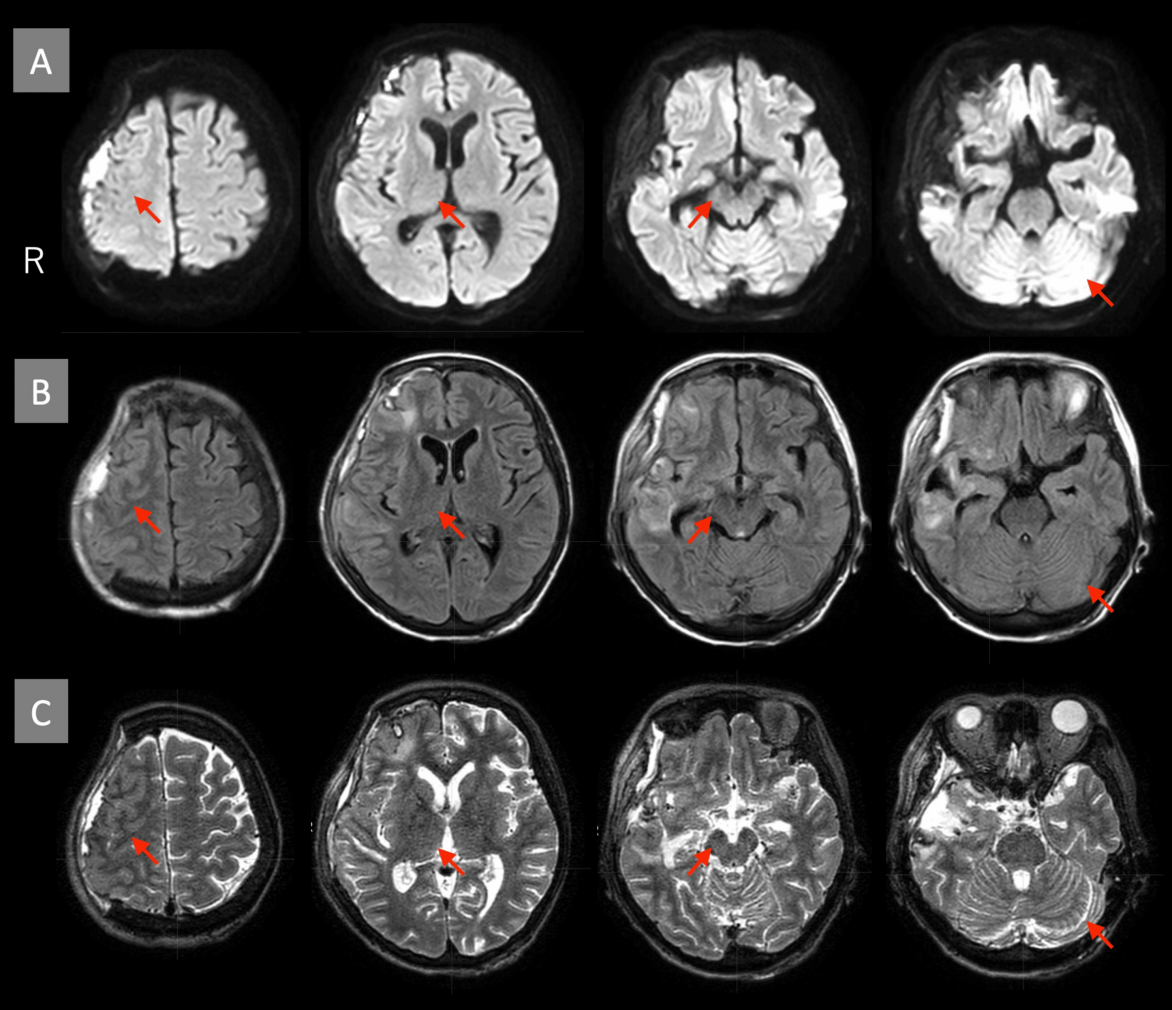
MRI of the head pre-cranioplasty (day 27) (A) Diffusion-weighted image (b=1000 s/mm²). (B) FLAIR image. (C) T2-weighted image. There were no lesions associated with motor, sensory, or coordination functions. The arrows show the right primary motor cortex, thalamus, cerebral peduncle, and left cerebellar hemisphere. MRI: magnetic resonance imaging; FLAIR: fluid-attenuated inversion recovery

On the 24th day of hospitalization, a neurological examination revealed slight weakness in the left upper and lower limbs. However, no significant motor paralysis or sensory deficits were observed. Coordination tests, including the finger-to-nose and finger-to-nose-to-finger tests, indicated mild dysmetria. Neuropsychological assessment revealed a score of 26 on the Mini-Mental State Examination (MMSE), and no evidence of limb-kinetic apraxia was observed during finger movement or imitation tasks (Figure 2A). In daily activities, the patient reported difficulties with fine motor tasks, such as fumbling when tying rope or buttoning, motor incoordination when wearing a mask, and dropping items when retrieving them from her pockets with the left hand (Figure 2B, Video 1). Brain MRI conducted on the 27th day (including diffusion-weighted, T2-weighted, T2*-weighted, and fluid-attenuated inversion recovery (FLAIR) imaging) showed no lesions associated with motor, sensory, or coordination functions (Figure 3).

**Video 1:** Pre-cranioplasty fine motor tasks

On the 28th day, the patient underwent cranioplasty, resulting in significant improvement in the motor function of the left upper limb, enabling practical use in daily activities (Figure 2C, Video 2). A follow-up neuropsychological assessment on the 35th day revealed an improvement in cognitive function, with the MMSE score increasing to 28 out of 30.

**Video 2:** Post-cranioplasty fine motor tasks

Diffusion tensor imaging

DTI was performed on a 1.5T Philips Ingenia MR Scanner (Philips Medical System, The Netherlands) using single-shot echo-planar imaging (EPI). DWI was performed with two b-values (b=0 s/mm² and b=800 s/mm²) applied along 15 non-collinear diffusion-sensitizing gradient directions. Imaging parameters included a repetition time (TR) of 3520 ms, an echo time (TE) of 92 ms, a flip angle of 90°, a field of view (FOV) of 230 mm, and a slice thickness and spacing between slices of 2.5 mm. The acquisition matrix was 96 × 94, and the pixel size was 1.597 × 1.597 mm². The echo train length was 47.

DTI data were analyzed using the Functional MRI of the Brain (FMRIB) Software Library (FSL) v6.0.7.9 (University of Oxford, UK). Image processing began by extracting b=0 s/mm² images, followed by brain tissue segmentation using the brain extraction tool 'BET' with a fractional intensity threshold of 0.5. The extracted brain region was used as the target area for DTI processing. FA, MD, and RD maps were generated using the 'DTIFit' tool, and FA images were aligned to the FSL standard brain (FMRIB58_FA_1mm) through both linear and non-linear transformations using 'FLIRT' and 'FNIRT' tools. The warp fields derived from these transformations were subsequently applied to align MD and RD images to the same standard brain.

For analysis, regions of interest (ROIs) were defined on the horizontal section of the MNI152_T1_1mm standard brain within FSL. ROIs were manually delineated using the Automated Anatomical Labeling (AAL) Atlas in MRIcroGL v10.14.6 (University of South Carolina, USA) and included the primary motor cortex, primary sensory cortex, premotor cortex, supplementary motor area, putamen, thalamus, internal capsule, cerebral peduncle of the midbrain, and cerebellar hemispheres.

Average FA, MD, and RD values for each ROI were calculated using the 'fslstats' tool. FA values range from 0 to 1, with higher values indicating greater fiber directionality. A decrease in FA values compared to the contralateral corresponding region suggests white matter damage. MD and RD values are expressed in diffusion coefficient units (mm²/s). Higher MD values indicate increased isotropic diffusion, suggesting white matter damage, while higher RD values reflect increased perpendicular diffusion around neural fibers, indicative of myelin damage.

Analysis results

**Figure 4:**
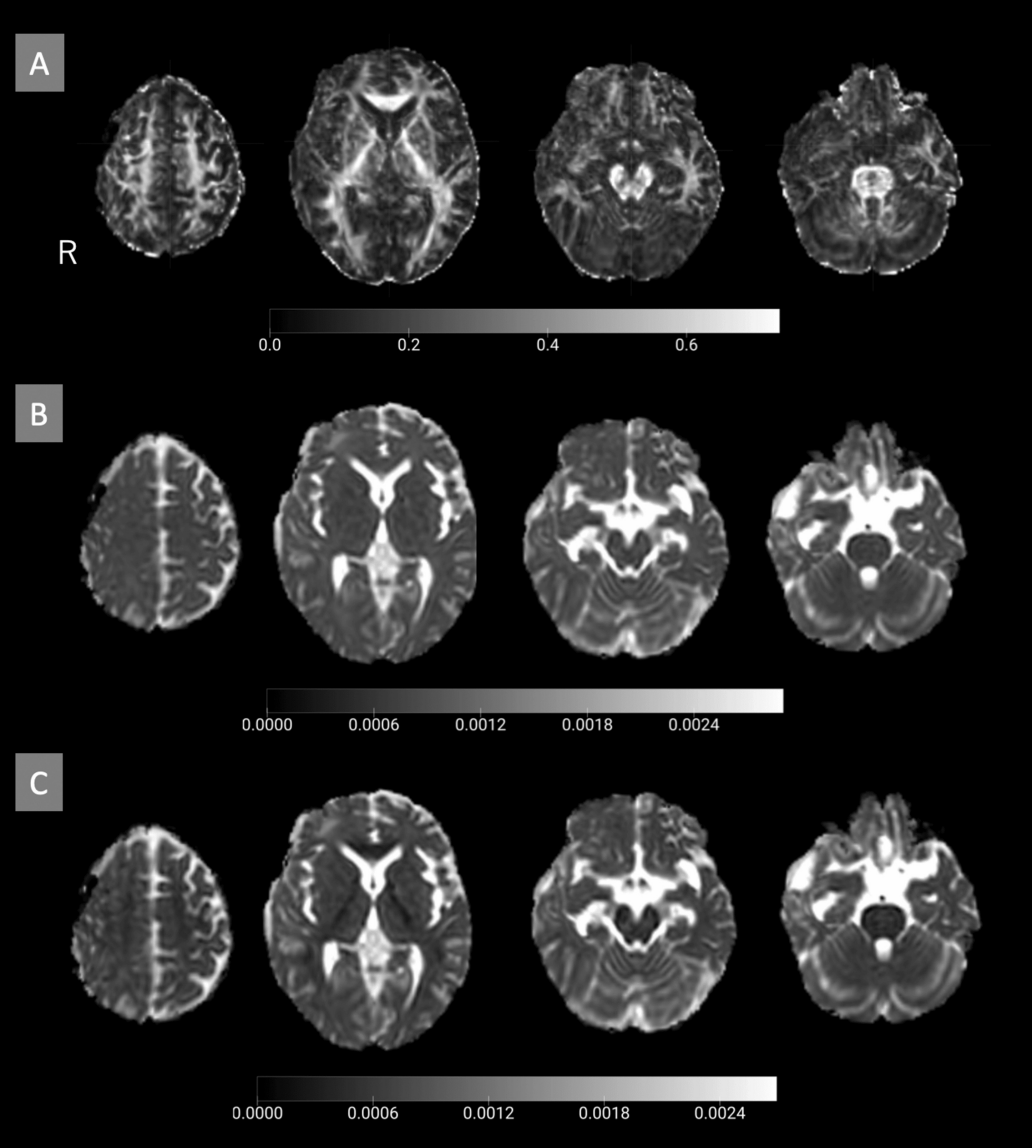
DTI-derived images pre-cranioplasty (day 27) (A) FA image. (B) MD image. (C) RD image DTI: diffusion tensor imaging; FA: fractional anisotropy; MD: mean diffusivity; RD: radial diffusivity

DTI scans were conducted on the 27th day of hospitalization, before cranioplasty, and on the 34th day, six days after the procedure. FA, MD, and RD images were aligned to the standard brain (Figure 4), and the average values of these indices were calculated for each ROI. 

In the primary motor cortex, FA values showed a notable increase (right (Rt): 1.51, left (Lt): 1.16), particularly on the right side, while MD (Rt: 1.08, Lt: 1.07) and RD (Rt: 1.02, Lt: 1.07) values remained unchanged. Similarly, in the premotor cortex, FA values showed a mild increase (Rt: 1.14, Lt: 0.96), whereas MD (Rt: 0.99, Lt: 1.06) and RD (Rt: 0.98, Lt: 1.06) values remained stable.

In the supplementary motor area, a slight increase in FA values was observed (Rt: 1.13, Lt: 1.06), while MD (Rt: 0.91, Lt: 0.98) and RD (Rt: 0.90, Lt: 0.98) values remained unchanged. The primary sensory cortex demonstrated a mild increase in FA values (Rt: 1.11, Lt: 0.94), while MD (Rt: 1.02, Lt: 1.06) and RD (Rt: 1.00, Lt: 1.07) showed no significant changes. 

No notable changes in FA, MD, or RD values were detected in the putamen, thalamus, internal capsule, cerebral peduncle of the midbrain, or cerebellar hemispheres (Table 1).

**Table 1:** Pre- and post-cranioplasty comparison of indices in various brain regions FA: fractional anisotropy; MD: mean diffusivity; RD: radial diffusivity; Rt: right; Lt: left

Region	FA (Rt)	FA (Lt)	MD (Rt)	MD (Lt)	RD (Rt)	RD (Lt)
Primary motor cortex	1.51	1.16	1.08	1.07	1.02	1.07
Premotor cortex	1.14	0.96	0.99	1.06	0.98	1.06
Supplementary motor area	1.13	1.06	0.91	0.98	0.90	0.98
Primary sensory cortex	1.11	0.94	1.02	1.06	1.00	1.07
Putamen	1.00	1.03	0.96	1.02	0.97	1.01
Thalamus	0.99	1.04	0.93	0.93	0.92	0.94
Internal capsule	1.03	0.94	0.97	1.01	0.96	1.05
Cerebral peduncle	0.99	1.05	1.06	1.02	1.06	0.97
Cerebellar hemispheres	1.06	1.03	0.96	0.99	0.96	0.99

## Discussion

This case of TBI with subsequent brain herniation demonstrated upper limb motor dysfunction despite the absence of evident lesions associated with motor or sensory functions on conventional MRI. Notably, significant improvements in neurological symptoms were observed following cranioplasty. Using pre- and post-cranioplasty DTI indices, including FA, MD, and RD values, we investigated the factors contributing to motor dysfunction and symptom improvement.

The changes in FA values observed in this study ranged from 0.94 to 1.51 times pre- to post-cranioplasty. While no established reference values for FA exist, previous studies have reported that increases of 1.06 to 1.3 times or greater are statistically and clinically significant [2,4,10]. The changes observed in several regions in this case are thought to reflect a recovery in the microstructural integrity of white matter fibers. Additionally, while changes in FA values may reflect structural alterations in myelin and axons [11], no significant changes were observed in RD values, which are indicative of myelin damage. This suggests that the myelin structure was likely preserved.

Decompressive craniectomy is a surgical intervention performed to manage elevated intracranial pressure. In this case, internal decompression involved resection of a portion of the right temporal lobe, while motor dysfunction is not generally expected due to the functional properties of the temporal lobe. Conversely, external decompression, which involves the removal of a portion of the skull, exposes brain tissue to atmospheric pressure. This exposure can result in reduced cerebral blood flow and the appearance of neurological symptoms. As reported in previous studies, cranioplasty has been shown to improve cerebral blood flow and alleviate neurological symptoms [12]. Similarly, in this case, neurological symptoms improved following cranioplasty, suggesting that changes in the intracranial environment contributed to the observed recovery.

DTI analysis revealed a significant increase in FA values, particularly in the right primary motor cortex, which correlated with improvements in upper limb motor function. In contrast, MD and RD values showed no significant changes pre- to post-cranioplasty, suggesting that the density and structure of white matter remained largely intact. These findings imply that the primary mechanism of the motor dysfunction was likely related to impaired neural transmission due to atmospheric pressure exposure. The reduction of pressure following cranioplasty may have improved fiber orientation and enhanced neural transmission efficiency, thereby contributing to the observed symptom recovery.

The observed fine motor dysfunctions of the upper limb, identified through neurological examinations and daily activity observations, were considered potentially related to sensory deficits or motor incoordination. While motor incoordination is commonly attributed to cerebellar or sensory area damage [13], no significant lesions were detected in these regions on conventional MRI, and no notable changes were observed in FA analysis of related areas. In this case, no paralysis or reduced speed was observed during simpler single-joint movements, but fine motor dysfunction was observed during tasks requiring more complex fine motor functions. This suggests that the significant FA increase observed in the primary motor cortex might reflect the fine motor dysfunctions caused by microstructural disruptions in neural transmission.

Nonetheless, this study has several limitations. First, despite the application of standard brain image transformations, brain morphology may not have been fully captured due to tissue loss resulting from internal decompression surgery and structural changes induced by cranioplasty. Second, manual ROI placement may have introduced errors or variability, potentially influencing the results. Finally, as this is a single case report, the findings are inherently limited in their generalizability. Further studies with larger sample sizes are required to validate these findings and elucidate the underlying mechanisms.

## Conclusions

In this case of TBI, where no apparent brain damage was observed in the motor and sensory areas, the patient presented with upper limb fine motor dysfunction. We investigated the underlying factors through the analysis of DTI data. The alleviation of symptoms following cranioplasty, coupled with significant changes in FA values pre- and post-cranioplasty, highlighted alterations in the primary motor cortex, a region crucial for motor coordination. These changes in the primary motor cortex were suggested to be related to the improvement of symptoms. This study indicates that DTI is a valuable tool for capturing subtle changes in brain structures that are difficult to assess with conventional imaging techniques.
